# Highly Efficient Mineralization of Typical PPCPs in Medical Wastewater via P25TiO_2_ Photocatalysis Under Sunlight Irradiation

**DOI:** 10.3390/molecules31071163

**Published:** 2026-03-31

**Authors:** Meiqi Gao, Xinyan Hou, Hongmei Li, Yansen Han, Jianing Wang, Yanqiu Cao

**Affiliations:** 1 School of Chemistry and Biological Engineering, University of Science and Technology Beijing, Beijing 100083, China; gaomq1211@163.com (M.G.);; 2 College of Life Science and Technology, Beijing University of Chemical Technology, Beijing 100029, China

**Keywords:** PPCPs, degradation, mineralization, medical wastewater, nanomaterials

## Abstract

Pharmaceuticals and personal care products (PPCPs), as persistent organic pollutants, are widely present in various aquatic environments. Their long-term presence in aquatic environments poses a potential threat to ecosystems and human health. This study established an efficient, green, and cost-effective photocatalytic method using P25 titanium dioxide (P25TiO_2_) to simultaneously degrade five representative PPCPs (methyl paraben (MeP), carbamazepine (CBZ), bisphenol A (BPA), diclofenac (DFC), and triclosan (TCS), while elucidating the reaction mechanisms. Under sunlight irradiation, degradation rates for all five PPCPs reached 100%, achieving near-complete mineralization with total organic carbon (TOC) removal rates exceeding 95%. This demonstrates the system’s exceptional capability to not only degrade the parent compounds but to thoroughly convert them into benign inorganic substances. We systematically investigated the effects of catalyst concentration, initial pollutant concentration, light intensity, pH, and various common inorganic anions (chloride, sulfate, bicarbonate, phosphate) and humic acid (HA) on the degradation process. Additionally, mechanistic studies indicated that hydroxyl radicals (·OH) are the primary active species in the system. The degradation rate differences among various persistent organic pollutants (DFC > BPA > TCS > CBZ > MeP) primarily stem from variations in the reactivity of different functional groups within their molecular structures toward ·OH. In summary, this study provides a promising and practical solution for treating complex medical wastewater containing five typical PPCPs.

## 1. Introduction

As a new category of pollutants, PPCPs are closely intertwined with daily life and industrial activities. PPCPs are diverse and complex in composition, encompassing human and veterinary pharmaceuticals, chemical consumer products, and additives used in manufacturing and processing [1]. Examples include antibiotics, anti-inflammatory drugs, antiviral medications, antihypertensives, analgesics, as well as personal care products such as cosmetics, hair dyes, shampoos, and fragrances [2]. Among these, MeP [3], CBZ [4], BPA [5], DCF [6], and TCS [7] are common and highly polluting aquatic contaminants. Due to their widespread use, these substances are frequently detected in medical wastewater and pose multifaceted threats to ecosystems [8]. For instance, DFC exhibits potent organ toxicity to fish [6]; CBZ, due to its structural stability, is extremely difficult to degrade, leading to persistent pollution; while BPA, MeP, and TCS are known endocrine disruptors capable of interfering with aquatic organisms hormonal balance, posing long-term ecological risks [9]. These substances enter the water cycle through multiple pathways, including domestic sewage, hospital wastewater, industrial discharges, and surface runoff [10]. Due to their structural stability and poor biodegradability, conventional medical wastewater treatment processes (such as the activated sludge method) often struggle to effectively remove them [11]. Consequently, these substances are frequently detected in rivers, lakes, and even drinking water sources, posing potential threats to aquatic ecosystems and potentially impacting human health through bioaccumulation in the food chain [12].

To address the challenges posed by PPCPs pollution, various water treatment technologies have been developed and applied. Physical methods like activated carbon adsorption and membrane filtration demonstrate high removal efficiency but essentially transfer pollutants from the liquid phase to the solid phase or concentrate, failing to achieve fundamental degradation. They also face secondary pollution issues such as adsorbent regeneration difficulties and membrane fouling, along with high costs. In contrast, advanced oxidation processes (AOPs), which focus on generating highly oxidative free radicals such as hydroxyl radicals (·OH) [13], are considered an effective approach to completely degrade such persistent organic pollutants [14]. However, many advanced oxidation processes report high degradation rates for parent pollutants but often neglect the crucial aspect of mineralization. The formation of persistent and sometimes more toxic intermediate products can pose a continued environmental risk. Therefore, achieving a high total organic carbon (TOC) removal rate is a critical indicator of a truly effective and environmentally safe treatment technology [15].

Among various AOPs, semiconductor-based multiphase photocatalytic technology has garnered significant attention due to its unique advantages [16]. This technology directly harnesses solar energy to completely mineralize organic pollutants into inorganic small molecules like CO_2_ and H_2_O under ambient temperature and pressure conditions, offering green, efficient, and secondary pollution-free characteristics. Among these, titanium dioxide (TiO_2_), particularly the commercial grade P25, is widely recognized as one of the most promising photocatalysts due to its high photocatalytic activity, chemical stability, low cost, and non-toxicity [17]. A large number of studies have confirmed the excellent performance of TiO_2_ in degrading individual PPCPs [18]. For example, Calza et al. [19]. reported in detail the efficient photocatalytic degradation effect of TiO_2_ on diclofenac.

However, PPCPs in real aquatic environments often exist as complex mixtures of multiple coexisting pollutants. Additionally, inorganic ions and natural organic matter present in the aquatic matrix can influence the degradation process. Despite the continuous development of novel photocatalytic materials, studies systematically investigating the degradation selectivity, kinetic differences, and performance of P25TiO_2_ catalysts in complex aquatic matrices when treating mixtures of structurally diverse PPCPs remain limited. Therefore, this study aims to systematically evaluate the performance of the P25TiO_2_ photocatalytic system in simultaneously degrading five representative PPCPs (MeP, CBZ, BPA, DFC, TCS). By optimizing key operational parameters, we investigate the degradation kinetics and intrinsic reaction mechanisms within the mixed system and assess the impact of common coexisting substances in water bodies. Provide scientific basis and data support for the practical application of this technology in treating medical wastewater contaminated with PPCPs.

## 2. Results and Discussion

### 2.1. Effect of Reaction Conditions on the Degradation of Five PPCPs

The activity of photocatalytic reactions is influenced by the external environment. The main external factors affecting photocatalytic reactions include the type and intensity of the light source, the amount of catalyst used, the initial pH value of the solution, and the co-existing substances in the solution [20,21].

#### 2.1.1. Photocatalyst Dosage

**Figure 1:**
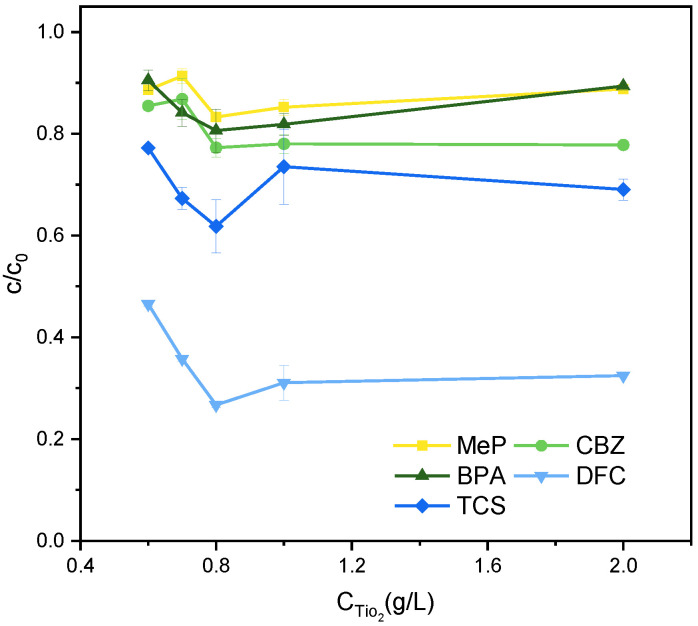
Effect of TiO_2_ Concentration on the Degradation of MeP, CBZ, BPA, DFC, and TCS. Reaction conditions: [MeP], [CBZ], [BPA], [DFC], [TCS] = 5 mg/L, T = 25°C, pH = 7, light intensity = 4.4 mW/cm^2^, reaction time 2 h.

Optimal photocatalyst dosage is critical for achieving ideal reaction conditions. As shown in Figure 1, the catalyst dosage was varied from 0.6 g/L to 2.0 g/L under a fixed mixed PPCPs concentration of 5 mg/L. Photocatalytic activity increased when the catalyst dosage rose from 0.6 g/L to 0.8 g/L. This increase resulted from the gradual rise in reactive sites as catalyst dosage increased, leading to greater radical generation. However, when the catalyst dosage exceeded 0.8 g/L, the degradation rate slightly decreased. This decline may be attributed to excessive catalyst hindering light scattering, causing photon quenching [22]. Therefore, the optimal dosage parameter for subsequent experiments was determined to be 0.8 g/L.

#### 2.1.2. Effect of Irradiance

**Figure 2:**
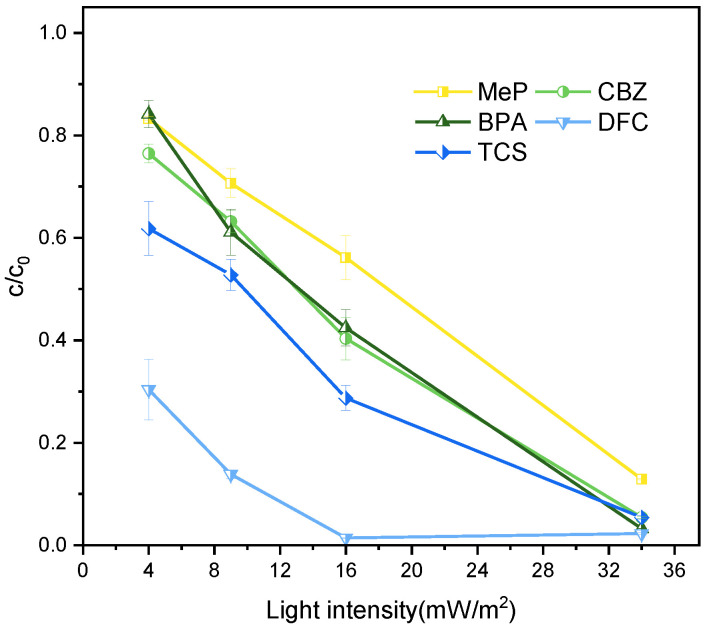
Effect of light intensity on the degradation of MeP, CBZ, BPA, DFC, and TCS. Reaction conditions: [MeP], [CBZ], [BPA], [DFC], [TCS] = 5 mg/L, T = 25°C, pH = 7, [TiO_2_] = 0.8 g/L, reaction time 2 h.

Light intensity is one of the core factors influencing UV photocatalytic reactions, affecting the degradation process by regulating the generation and utilization efficiency of photogenerated carriers. According to the photocatalytic reaction mechanism, UV light of appropriate intensity can excite the catalyst TiO_2_ to generate electron-hole pairs, driving the occurrence of redox reactions. However, higher light intensity is not always better. At excessively low intensities, insufficient photon energy input results in limited electron-hole pair generation, restricting reaction rates and reducing degradation efficiency. Conversely, excessively high intensities may trigger excessive carrier recombination, catalyst photocorrosion, light saturation, or cumulative thermal effects within the system, all of which diminish degradation efficiency. As shown in Figure 2, degradation outcomes vary significantly with different light intensities. The UV intensity referenced here denotes the average value observed in the Northern Hemisphere between 10:00 am and 4:00 pm daily. Specifically, the average UV intensity on the winter solstice (the shortest daylight period of the year) is approximately 16 mW/cm^2^ (As shown in Table 1), while that on the summer solstice (the longest daylight period) is approximately 34 mW/cm^2^. In practical applications, 16 mW/cm^2^ represents a UV intensity closer to the minimum levels found in natural environments. Therefore, choosing 16 mW/cm^2^ as the experimental condition not only better simulates the intensity of ultraviolet light in actual environments, but also ensures that the experimental results are more representative and universal.

#### 2.1.3. Effect of Initial pH

**Figure 3:**
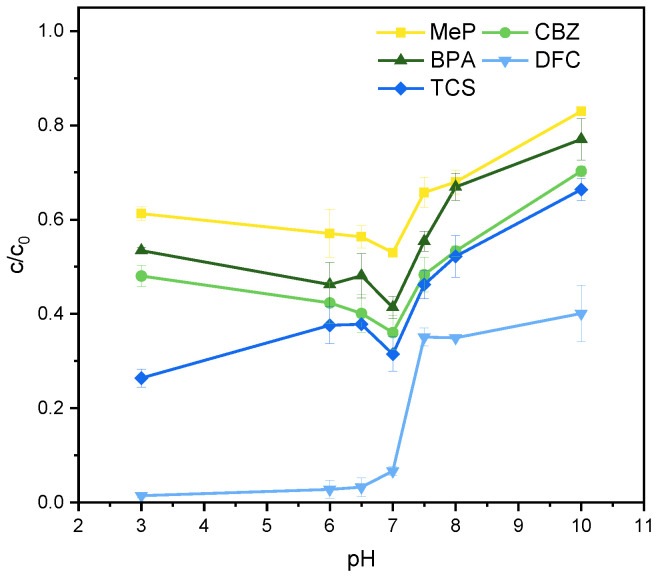
Effect of pH on the degradation of MeP, CBZ, BPA, DFC, and TCS. Reaction conditions: [MeP], [CBZ], [BPA], [DFC], [TCS] = 5 mg/L, T = 25°C, [TiO_2_] = 0.8 g/L, reaction time 2 h, light intensity = 16 mW/cm^2^.

In the TiO_2_ photocatalytic degradation system for MeP, CBZ, BPA, DFC, and TCS, pH significantly influences radical generation and pollutant degradation efficiency. As shown in Figure 3, at pH 7, the residual rates of all five PPCPs were low, indicating excellent degradation performance. At this pH, the effective generation of active radicals such as ·OH is ensured, while adsorption further promotes pollutant degradation. Therefore, pH = 7 was selected as the optimal pH for this study, and subsequent experiments were conducted under these conditions.

#### 2.1.4. Effects of Coexisting Anions and Humic Acid

In actual water bodies, background solutions often contain substantial amounts of inorganic anions and various dissolved organic compounds such as natural humic acids. The presence of these substances competes with the free radicals generated in photocatalytic reaction systems, thereby affecting the degradation efficiency of target pollutants. This study evaluated the effects of five common coexisting anions (Cl^−^, SO_4_^2−^, HCO_3_^−^, H_2_PO_4_^−^) and a typical natural organic compound (HA) on the removal of MeP, CBZ, BPA, DFC, and TCS by the reaction system.

**Figure 4:**
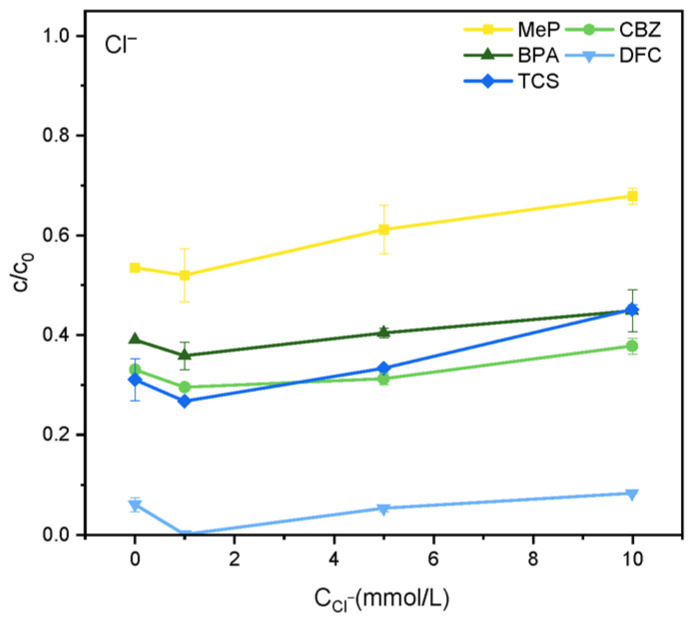
Effect of Cl^−^ on the degradation of MeP, CBZ, BPA, DFC, and TCS.

**Figure 5:**
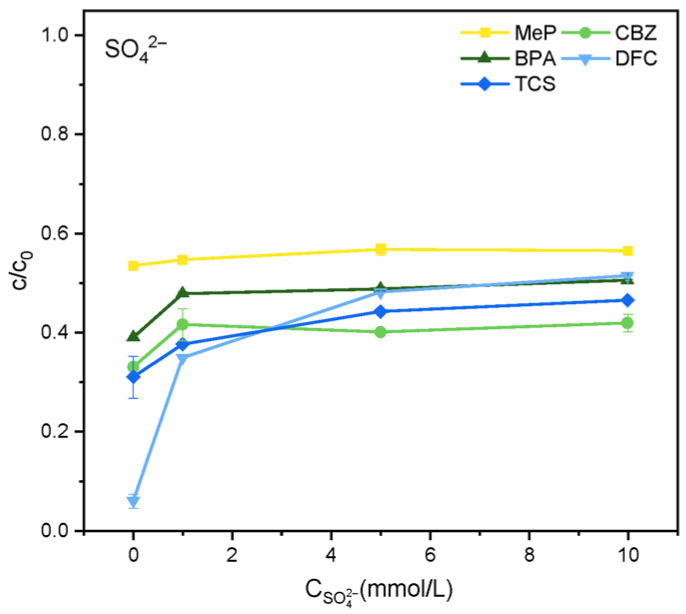
Effect of SO_4_^2−^ on the degradation of MeP, CBZ, BPA, DFC, and TCS.

As shown in Figure 4, at lower Cl^−^ concentrations, the degradation efficiencies of MeP, CBZ, BPA, DFC, and TCS increased to some extent. At a Cl^−^ concentration of 1 mmol·L^−1^, the degradation efficiency of MeP increased from 46% to 49% within 2 h, CBZ from 64% to 71%, and BPA from 61% to 65%, DFC increased from 94% to 99%, and TCS increased from 69% to 74%. However, when the Cl^−^ concentration was increased to 5 mmol·L^−1^ and 10 mmol·L^−1^, the degradation of MeP, CBZ, BPA, DFC, and TCS was significantly inhibited. The primary reason for the increased degradation rates of the five substances at low Cl^−^ concentrations is that Cl^−^ can react with ·OH, as shown in Equation (1), and the redox potential of the generated chlorine radical (Cl·/Cl^−^) is 2.4 V, endowing it with high oxidative capacity. However, excess Cl^−^ reacts with Cl· to form the less reactive chlorine radical ${\mathrm{Cl}}_{2}^{-}$· (2) [23], whose redox potential is only 1.36 V, thereby inhibiting degradation. As shown in Figure 5, SO_4_^2−^ partially inhibits the degradation of five organic pollutants within 2 h. This may occur because SO_4_^2−^ competes with organic pollutants for oxidation reactions with h^+^ or ·OH, ultimately forming SO_4_^2−^ and thereby suppressing degradation, as depicted in Reactions (3) and (4) [24].

(1) $${\mathrm{Cl}}^{-}+\cdot \mathrm{OH}\;\to \;{\mathrm{OH}}^{-}+\mathrm{Cl}\cdot$$

(2) $${\mathrm{Cl}}^{-}+\mathrm{Cl}\cdot \;\to \;\;\;{\mathrm{Cl}}_{2}^{-}\cdot$$

(3) $${{\mathrm{SO}}_{4}}^{2-}+\cdot \mathrm{OH}\to \;{\mathrm{OH}}^{-}+{{\mathrm{SO}}_{4}}^{-}$$

(4) $${{\mathrm{SO}}_{4}}^{2-}+h^{+}\to \;{{\mathrm{SO}}_{4}}^{-}$$

**Figure 6:**
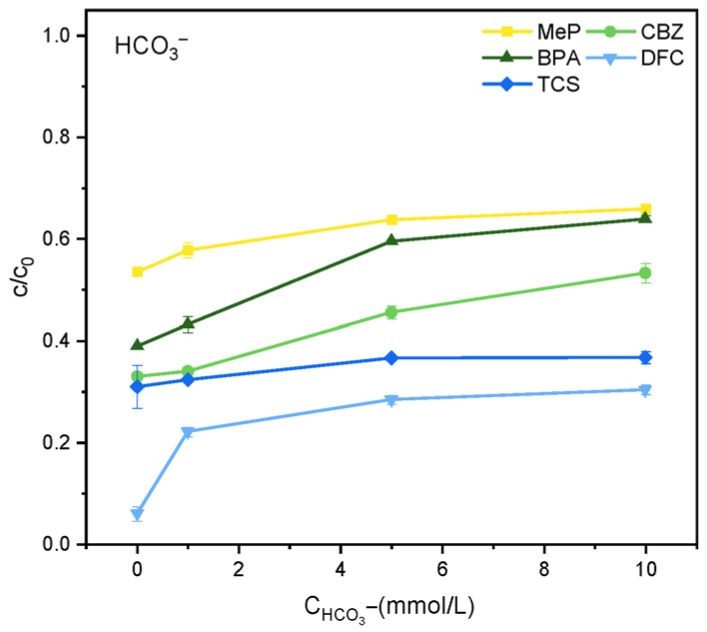
Effect of HCO_3_^−^ on the degradation of MeP, CBZ, BPA, DFC, and TCS.

**Figure 7:**
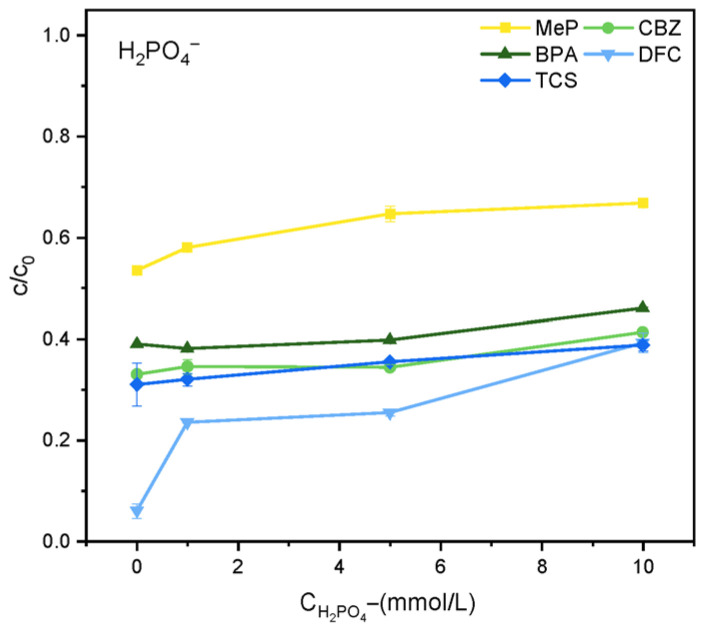
Effect of H_2_PO_4_^−^ on the degradation of MeP, CBZ, BPA, DFC, and TCS.

The effect of HCO_3_^−^ on degradation efficiency is shown in Figure 6. When the reaction has been ongoing for 2 h, as the concentration of HCO_3_^−^ increases, the degree of reaction inhibition gradually increases. This is primarily because HCO_3_^−^ reacts with ·OH to form the less reactive HCO_3_·, thereby reducing degradation efficiency. Additionally, HCO_3_^−^ consumes ·OH generated in the reaction system, converting it to CO_3_^2−^/HCO_3_^−^ with a lower redox potential. This, coupled with CO_3_^2−^/HCO_3_^−^ competing with other free radicals for reaction with organic pollutants, further reduces the degradation rate. Similarly, Figure 7 shows that different concentrations of H_2_PO_4_^−^ also inhibit the reaction system within 2 h. This is primarily due to the reactivity of H_2_PO_4_^−^ with ROS (·OH), which readily converts it into the weakly oxidizing H_2_PO_4_·, thereby reducing degradation efficiency.

(5) $$h^{+}+\;{\mathrm{OH}}^{-}\;\to \;\cdot \mathrm{OH}$$

(6) $${{\mathrm{HCO}}_{3}}^{-}+\cdot \mathrm{OH}\;\to \;{\mathrm{CO}}_{3}\cdot^{-}+\;H_{2}O$$

(7) $${{\mathrm{HCO}}_{3}}^{-}+\;h^{+}\;\to \;{\mathrm{HCO}}_{3}\cdot^{-}$$

(8) $${{\mathrm{CO}}_{3}}^{2-}+\cdot \mathrm{OH}\;\to \;{\mathrm{CO}}_{3}\cdot^{-}+\;{\mathrm{OH}}^{-}$$

**Figure 8:**
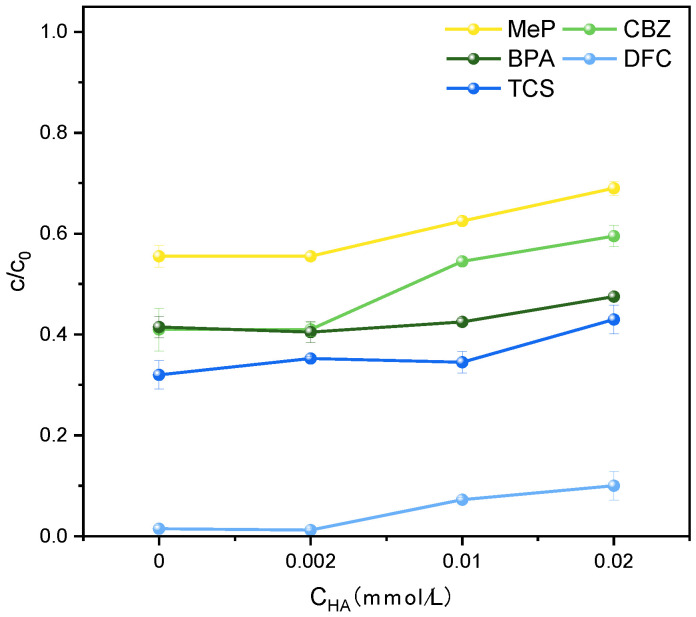
Effect of HA on the degradation of MeP, CBZ, BPA, DFC, and TCS.

As evident from Figure 8, HA significantly inhibits the degradation of all five PPCPs within 2 h. The inhibitory effect increases with higher initial HA concentrations. This may result from quenching reactions between HA and partially generated ·OH radicals in the system, thereby reducing degradation efficiency. Previous studies support this mechanism.

**Figure 9:**
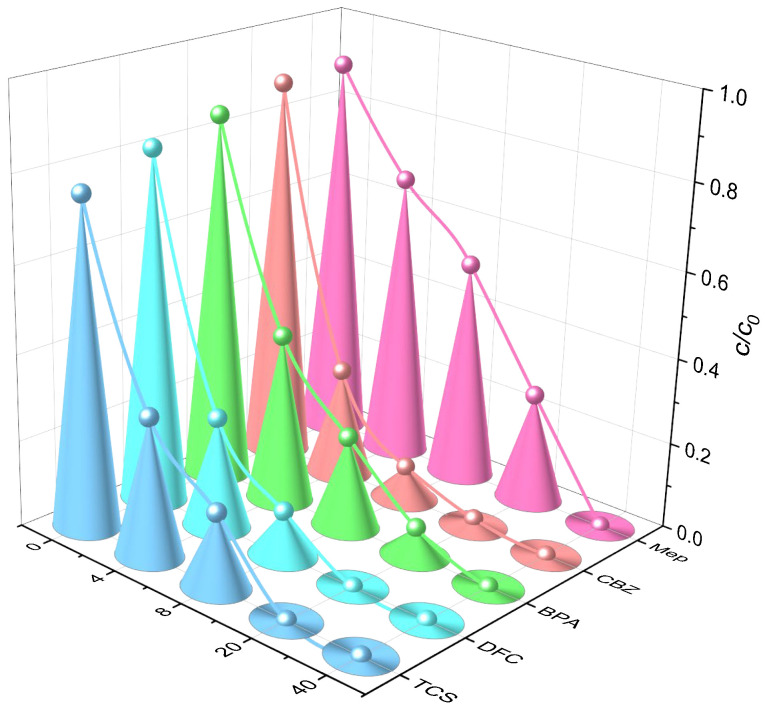
Effect of multiple coexisting ions on the degradation of 5 PPCPs.

Figure 9 illustrates the degradation of the five compounds under the presence of various interfering ions, with their respective concentrations being [Cl^−^], [SO_4_^2−^], [HCO_3_^−^], [H_2_PO_4_^−^] = 10 mmol/L, and [HA] = 0.02 mmol/L. Even at higher concentrations of interfering ions, all substances ultimately achieved complete degradation as reaction time increased.

#### 2.1.5. Analysis of Catalyst Stability

**Figure 10:**
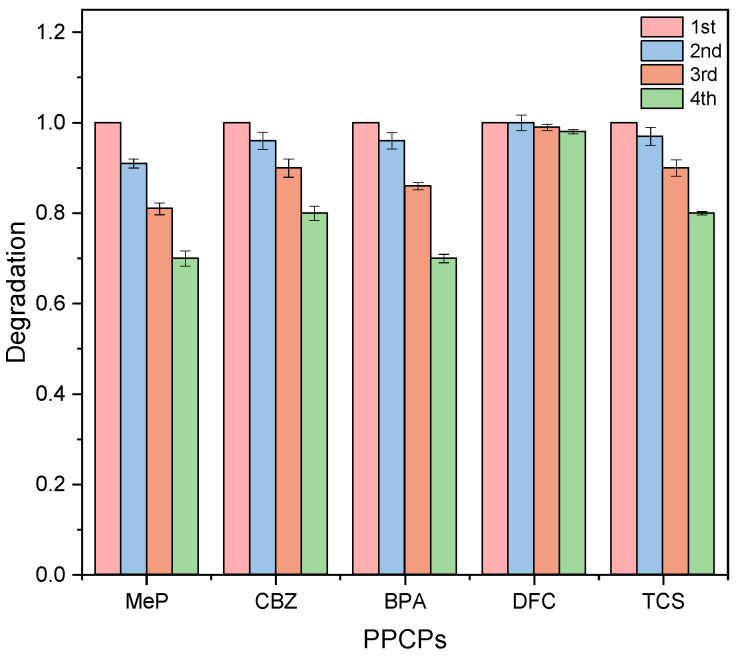
Effect of the number of cycles on the degradation of five PPCPs.

The reusability and stability of a catalyst are key factors in evaluating its economic viability in practical applications. Therefore, under identical test conditions, the reusability of P25TiO_2_ was assessed by comparing the removal rates of five PPCPs across four cycles of degradation testing. As shown in Figure 10, catalytic performance declined slightly after four consecutive runs. This may be attributed to catalyst loss or the coverage of active sites by pollutants and their degradation intermediates, which could not be completely removed by washing, resulting in a reduction in active material and, consequently, a decrease in catalytic performance. However, after four cycles, the degradation rates of the five PPCPs remained above 70% within 8 h, demonstrating good cycling stability.

### 2.2. Simultaneous Degradation of Five PPCPs Under Different Light Sources

#### 2.2.1. Simultaneous Degradation of Five PPCPs via UV Photocatalysis

Previous studies indicate that the optimal conditions for this research are: P25TiO_2_ concentration of 0.8 g/L, light intensity of 16 mW/cm^2^, and initial pH of 7. Under these conditions, simultaneous degradation of five PPCPs was conducted. Figure 10 demonstrates the degradation efficiency of catalyst P25TiO_2_ for the five PPCPs under optimal conditions. After 8 h, MeP, CBZ, BPA, DFC, and TCS, all five PPCPs were completely degraded, with a degradation rate of 100%.

**Figure 11:**
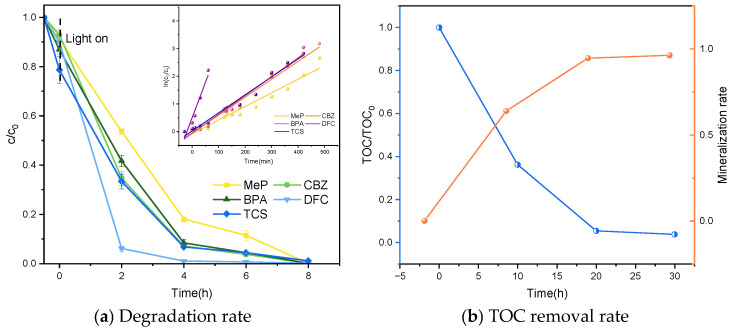
Degradation and mineralization of five PPCPs under UV photocatalysis.

To investigate the mineralization rates of P25TiO_2_ for the simultaneous degradation of MeP, CBZ, BPA, DFC, and TCS, the TOC removal rates during the degradation reaction were monitored, as shown in Figure 11. The figure indicates that the mineralization rate continuously increased with reaction progression. After 0.5 h of dark reaction followed by 30 h of photocatalytic reaction, the mineralization rate of the mixed sample containing all five PPCPs reached 96.4%. This demonstrates the method’s significant degradation capability for treating complex pollutant mixtures. Additionally, individual degradation experiments were conducted for each compound, and their respective mineralization rates were measured. As shown in Figure 6, the mineralization rates for MeP, CBZ, BPA, DFC, and TCS were 96.4%, 96.8%, 98.0%, 95.0%, and 97.3%, respectively. All individually degraded compounds achieved mineralization rates exceeding 95.0%, further validating the method’s effectiveness and reliability for treating single pollutants. These results indicate that the method is applicable not only to the degradation of complex pollutants but also to the treatment of single pollutants, demonstrating broad application prospects.

#### 2.2.2. Degradation Under Xenon Lamp

This study investigates the use of sunlight, a natural and inexhaustible light source, as a substitute for ultraviolet lamps in photocatalytic degradation experiments, exploring the economic feasibility and applicability of photocatalytic degradation of pollutants. Compared to artificial light sources, sunlight offers greater economic viability while enabling pollutant degradation without additional energy consumption. Therefore, this section employs sunlight as the light source in combination with photocatalysts to degrade five PPCPs, establishing a research foundation for pollutant degradation under natural light.

**Figure 12:**
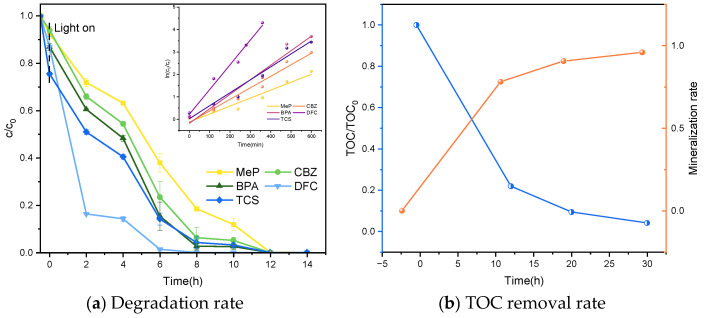
Degradation and mineralization of five PPCPs under xenon lamp.

Under simulated sunlight, using a xenon lamp as the light source, the system evaluated its degradation and mineralization on five typical organic pollutants: MeP, CBZ, BPA, DFC, and TCS. As shown in Figure 12, under conditions of 0.8 g L^−1^ catalyst dosage and pH 7, a mixed solution with initial concentrations of 5 mg L^−1^ for all five target compounds achieved 100% degradation after 12 h of continuous irradiation. Further mineralization rate testing (Figure 12) revealed that the mixed solution of five pollutants achieved a 96.0% mineralization rate after photocatalytic degradation. This mineralization rate indicates that the method not only effectively degrades organic pollutants but also significantly converts them into harmless small molecules such as CO_2_ and H_2_O.

#### 2.2.3. Degradation Under Sunlight

**Figure 13:**
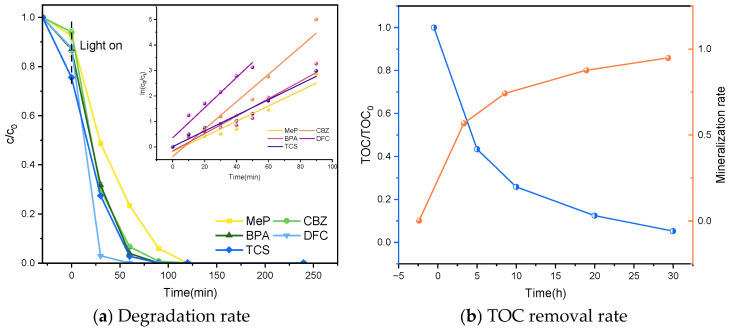
Degradation and mineralization of five PPCPs under Sunlight irradiation.

In this study, the performance of the photocatalytic degradation reaction was further validated under real sunlight irradiation. Experimental results demonstrate that the photocatalytic system utilizing sunlight as the light source exhibits significant efficacy in degrading multiple organic pollutants. Figure 13 illustrates the degradation of five PPCPs, showing that all samples achieved 100% degradation rate within a 2 h reaction time, with total organic carbon (TOC) removal rates achieving 95.0%, indicating the photocatalytic system high degradation efficiency under natural light conditions. Compared with traditional artificial light sources such as xenon lamps, sunlight, as an environmentally friendly and sustainable light source, can not only provide broad-spectrum lighting conditions but also significantly reduce the energy consumption of experiments, offering broader practical application prospects.

#### 2.2.4. Comparison of Photocatalytic Performance with Other Photocatalysts

As shown in Table 2, while several studies report high degradation percentages for individual PPCPs, their corresponding TOC removal rates are often significantly lower, typically ranging from 40% to 80%. For instance, Duan et al. achieved 89.85% degradation of Mep but only 40.12% TOC removal. In stark contrast, our work demonstrates both 100% degradation and over 95% mineralization for a complex mixture of five PPCPs, highlighting the superior efficiency and completeness of the P25TiO_2_ system.

### 2.3. Mechanistic

#### 2.3.1. Identification of Reactive Oxygen Species (ROS)

**Figure 14:**
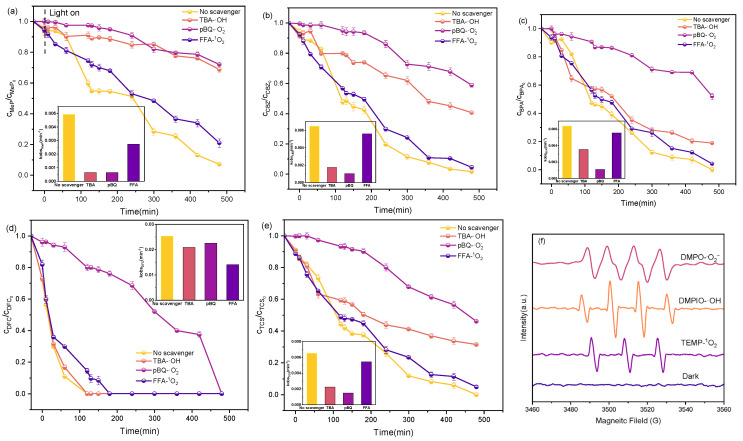
(**a**–**e**) Effects of different quenchers on the degradation of Mep, CBZ, BPA, DFC, and TCS; (**f**) Electron spin resonance (ESR) spectra of ·OH, $O_{2}^{-}$·, and ^1^O_2_ capture.

EPR spectroscopy and quenching experiments identified ROS species involved in the degradation of five PPCPs in the P25TiO_2_ system. First, using TEMP as a spin trap, the signal of TEMP^−1^O_2_ confirmed that ^1^O_2_ is also generated in the P25TiO_2_ system [48], as shown in Figure 14f. To further validate the presence of ^1^O_2_ in the system, a series of quenching experiments were conducted using FFA to scavenge ^1^O_2_. As shown in Figure 14a–e, even at 50 mM, FFA exhibited negligible inhibition of the removal of the five PPCPs, indicating that ^1^O_2_ contributes minimally to the removal of the five PPCPs. These results indicate that ^1^O_2_ is not the primary ROS in the P25TiO_2_ system.

To further investigate reactive species in the P25TiO_2_ system, EPR spectroscopy and quenching experiments were employed to detect $O_{2}^{-}$· and ·OH. DMPO was used as the spin trap for $O_{2}^{-}$· spin trap for EPR analysis to confirm the presence of $O_{2}^{-}$· [49]. A strong signal for the DMPO- $O_{2}^{-}$· adduct was observed in the EPR spectrum (Figure 14f), indicating the existence of $O_{2}^{-}$·. The presence of ·OH was verified via EPR analysis using DMPO as a spin trapper. As shown in Figure 14f, an EPR signal for DMPO-OH was observed in the reaction system, confirming the existence of ·OH. Concurrently, quenching experiments with pBQ and TBA further determined the contributions of $O_{2}^{-}$· and ·OH in the removal of five PPCPs. As shown in Figure 14a–e, pBQ (20 millimoles) inhibited the removal process of all five persistent organic pollutants, and at the same time led to the partial disappearance of $O_{2}^{-}$· and ·OH. Moreover, the addition of TBA exhibited some inhibitory effect on the removal of the five PPCPs. This indicates that in theP25TiO_2_ system, both $O_{2}^{-}$· and ·OH contribute to the removal of the five PPCPs, with ·OH being the primary ROS responsible for their removal.

#### 2.3.2. Kinetic Simulation

**Figure 15:**
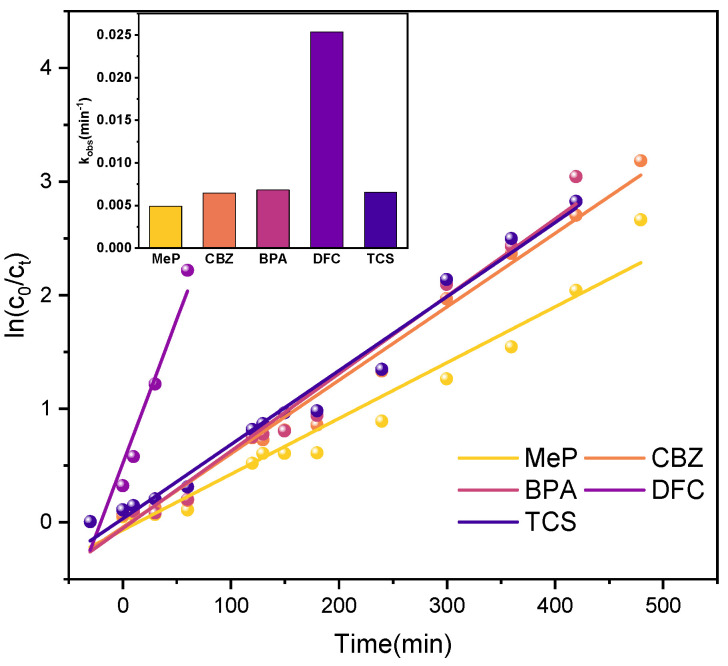
Kinetic simulation curves of 5 PPCPs (UV lamp).

Under UV irradiation, kinetic simulations were conducted for the degradation reactions of the five PPCPs. As shown in Figure 15, the kinetic fitting curves confirm that the UV-photocatalytic degradation of all five PPCPs follows pseudo-first-order kinetics.

−*ln(c_t_*/*c*_0_*)* = *kt* (9)

Here, k and t represent the kinetic constant and reaction time, respectively. Table 3 presents the reaction kinetic analysis of the P25TiO_2_ photocatalyst degrading MeP, CBZ, BPA, DFC, and TCS, including the reaction kinetic equation, rate constant k, and correlation coefficient R^2^. The correlation coefficients R^2^ were all above 0.96, indicating good correlation.

**Figure 16:**
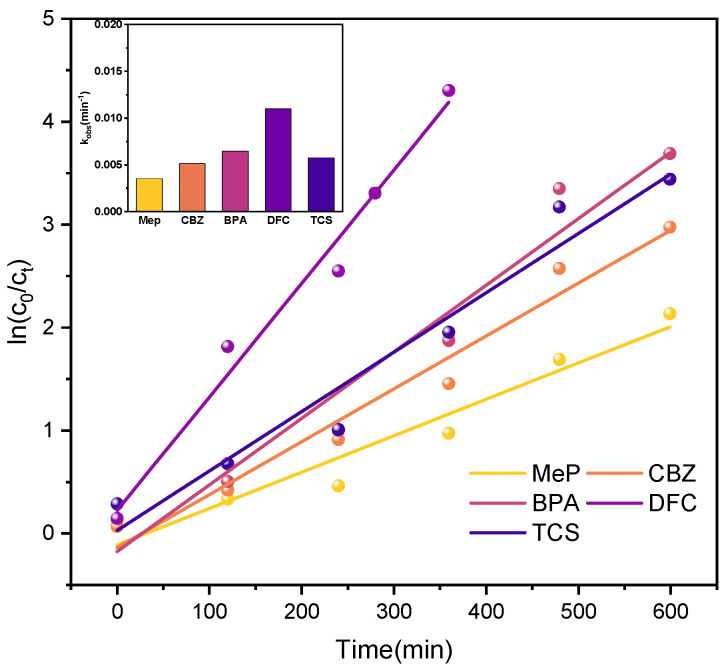
Kinetic simulation curves of five PPCPs (xenon lamp).

Figure 16 displays the kinetic fitting results for the reaction under xenon lamp irradiation, while Table 4 lists the kinetic parameters for the five PPCPs. Analysis of the degradation data indicates that the reaction follows a pseudo-first-order kinetic model. This demonstrates that the catalytic system exhibits excellent reactivity and stability under conventional conditions, possessing strong catalytic potential.

**Figure 17:**
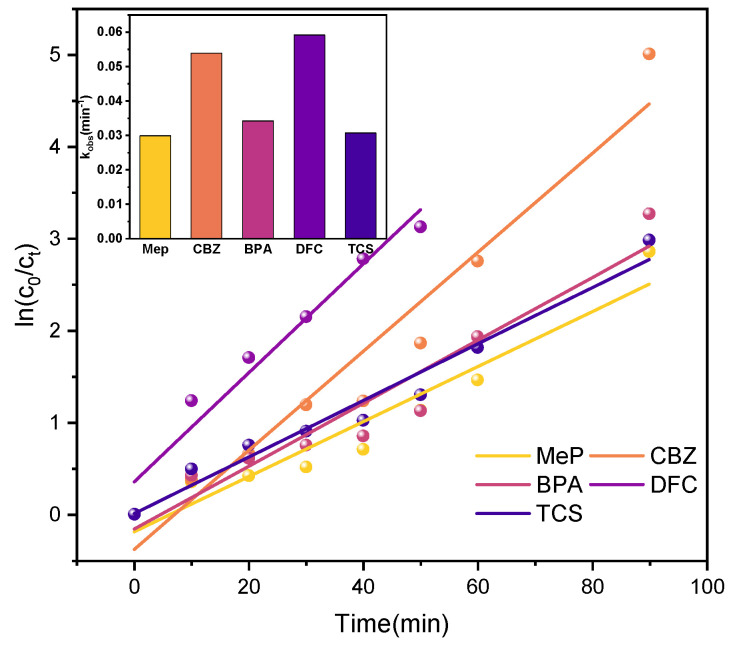
Kinetic simulation curves of 5 PPCPs (sunlight).

Under real sunlight irradiation, further kinetic analysis (Figure 17 and Table 5) indicates that the degradation process follows a pseudo-first-order kinetic model. Compared to xenon lamp illumination, using sunlight as the light source not only reduces experimental energy consumption but also better simulates pollutant degradation processes in natural environments during practical applications.

#### 2.3.3. Competitive Mechanism

The kinetic fitting results indicate that under UV light, xenon lamp, and visible light conditions, the degradation kinetics of the five target PPCPs in the P25TiO_2_ photocatalytic system exhibit significant differences. Their apparent rate constants k follow the order DFC > BPA > TCS > CBZ > MeP. In heterogeneous photocatalytic systems, the overall reaction rate is typically determined synergistically by two processes: mass transfer and adsorption of pollutants onto the catalyst surface, followed by subsequent surface chemical reactions. Therefore, explaining the observed rate differences requires comprehensive consideration of both aspects.

First, the adsorption process is jointly influenced by the surface charge of the catalyst and the molecular properties of the pollutant. TiO_2_ (as an n-type semiconductor photocatalyst with a bandgap of approximately 3.2 eV) generates photoexcited e^−^- h^+^ pairs upon light excitation, leading to the formation of ·OH, $O_{2}^{-}$· radicals. These reactive species are the core agents driving pollutant degradation. Concurrently, the charge state on the TiO_2_ surface directly influences pollutant adsorption behavior. Under the pH = 7 conditions of this study, the P25 TiO_2_ surface (pzc ≈ 6.3) carries a slight negative charge. Although this may induce electrostatic repulsion toward the anionic forms of similarly negatively charged compounds like DFC (pKa = 4.1), TCS (pKa = 7.9), and BPA (pKa = 9.6), experimental results indicate that DFC exhibits the fastest degradation rate [50]. This suggests that electrostatic interactions are not the sole or dominant factor determining the overall reaction rate. Furthermore, no clear linear relationship was observed between degradation rates and molecular hydrophobicity (LogK_ow_) or molecular size [51,52]. These phenomena indicate that while adsorption is a necessary prerequisite for the reaction, it is not the dominant factor determining overall rate differences. This suggests that the chemical reaction steps following surface adsorption are more critical.

Therefore, a more reasonable explanation is that the chemical reaction on the catalyst surface constitutes the rate-determining step for the entire degradation process. In the TiO_2_ photocatalytic system, hydroxyl radicals (·OH) serve as the primary strong electrophilic oxidizing species, and their attack efficiency is closely related to the distribution of electron density on the target molecule. Based on this analysis, the key reason DFC exhibits the fastest degradation rate lies in the secondary amine group (-NH-) within its structure. This group acts as a highly potent electron donor. Through the conjugation effect, it significantly increases the electron density of the two benzene rings, making them highly susceptible targets for ·OH attack. The reaction rate constant approaches the diffusion-controlled limit, completely overpowering the passivation effects of electron-withdrawing groups like chlorine and carboxyl groups within its own structure. BPA have high reactivity stems from its two phenol-activated benzene rings, providing abundant attack sites for ·OH. In contrast, TCS exhibits a slightly slower rate than BPA because it possesses only one phenol-activated benzene ring, while the other ring is passivated by three strongly electron-withdrawing chlorine atoms [53]. CBZ having degradation activity primarily originates from the lone pair on its central C=C double bond, an electron-rich site. However, its reactivity is lower than that of BPA and TCS, which possess multiple activated benzene rings [54]. Finally, MeP exhibits the slowest degradation rate. Its benzene ring is simultaneously influenced by the electron-donating -OH group and the strongly electron-withdrawing ester group (-COOCH_3_) [55], significantly weakening the overall electron density of the benzene ring and thereby reducing its reactivity with ·OH radicals.

In summary, the observed degradation rate order (DFC > BPA > TCS > CBZ > MeP) is not dominated by physical adsorption processes but is determined by the distribution of electron density within each pollutant’s molecular structure, ultimately dictated by their reactivity with ROS such as ·OH.

## 3. Materials and Methods

### 3.1. Reagents and Chemicals

Reference Standards: Methyl paraben (purity ≥ 99%), Carbamazepine (purity ≥ 99%), Bisphenol A (purity ≥ 99%), Diclofenac (purity ≥ 99%), Triclosan (purity ≥ 99%), supplied by Thermo Fisher Scientific (Waltham, MA, USA). Solvents: n-Octanol (AR, purity ≥ 99%), n-Heptanol (AR, purity ≥ 99%), iso-Octanol (AR, purity ≥ 99%), n-Pentanol (ACS grade) provided by Shanghai Aladdin Biochemical Technology Co., Ltd. (Shanghai, China). n-Hexanol (HPLC, purity ≥ 99.5%) provided by Beijing Marite Technology Co., Ltd. (Beijing, China). Methanol (HPLC/ACS grade) and acetonitrile (HPLC/ACS grade) were supplied by Beijing Bailingwei Technology Co., Ltd. (Beijing, China). Salts: Sodium chloride (purity ≥ 99%) was supplied by Sango Bioengineering Co., Ltd. (Shanghai, China). Anhydrous sodium dihydrogen phosphate (purity ≥ 99%), magnesium sulfate (purity ≥ 99%), and sodium bicarbonate (purity ≥ 99%) were supplied by Beijing Bailingwei Technology Co., Ltd. Others: Humic acid (purity ≥ 98%) was supplied by Shanghai McLean Biochemical Technology Co., Ltd. (Shanghai, China). Sodium hydroxide (purity ≥ 98%) and glacial acetic acid (purity ≥ 98%) were supplied by Beijing Bailingwei Technology Co., Ltd. P25 titanium dioxide (purity ≥ 99%) was supplied by Beijing Inokai Technology Co., Ltd. (Beijing, China). Ultrapure water was prepared in the laboratory.

### 3.2. Photodegradation Experiment

This study’s photocatalytic degradation experiment was divided into two parts: optimization of reaction conditions and investigation of degradation performance under different light sources. The selection of light sources and experimental procedures are as follows: in the optimization stage of reaction conditions, the light source was provided an 80 W ultraviolet lamp emitting at 365 nm. In the photocatalytic reaction experiments, P25TiO_2_ was dispersed in 40 mL of a mixed solution containing MeP, CBZ, BPA, DFC, and TCS (5 mg L^−1^), followed by magnetic stirring in the dark for 30 min to achieve adsorption–desorption equilibrium. During irradiation, 1 mL of reaction solution was extracted at specific time intervals and immediately filtered for analysis. The residual concentrations of the five PPCPs were determined using high-performance liquid chromatography (HPLC, Shimadzu Co., Tokyo, Japan). Subsequently, optimization experiments were conducted for photocatalyst dosage (0.6–2.0 g/L), light intensity (4.4–34 mW/cm^2^), and pH (3–10). Additionally, interference from coexisting ions and organic matter in water was evaluated.

After optimizing the reaction conditions, photocatalytic degradation experiments were conducted using a 500 W xenon lamp (200–2000 nm) under optimal conditions, following the same experimental procedure as in the condition optimization experiments.

In the natural sunlight experiments, P25 TiO_2_ was dispersed in 40 mL of a mixed solution (containing 5 types of PPCPs at a concentration of 5 mg·L^−1^). The mixture was stirred under dark conditions using a magnetic stirrer for 30 min to achieve adsorption–desorption equilibrium, after which it was exposed to natural sunlight. Sunlight degradation experiments were conducted in June and December, respectively. Samples of 1 mL were collected at predetermined time intervals, immediately filtered, and analyzed.

After optimizing the optimal reaction conditions, further degradation performance exploration experiments were conducted under different light sources. Xenon lamps were used to simulate sunlight and natural light as driving sources, and the degradation effects of the target PPCPs were investigated.

### 3.3. Analytical Methods

This study employed high-performance liquid chromatography with a diode array detector (HPLC-DAD) to detect MeP, CBZ, BPA, DFC, and TCS. Separation was achieved using a Diamonsil Plus C18 column (250 × 4.6 mm, 5 μm). The column temperature was maintained at 45 °C. The mobile phase consisted of methanol and 4% glacial acetic acid. The injection volume was 10 μL, and the flow rate was 1.0 mL/min. A binary high-pressure gradient elution program was employed: 0–9 min: methanol—4% glacial acetic acid (66/34, *v*/*v*), 9–18 min: methanol—4% glacial acetic acid (90/10, *v*/*v*), 18–24 min: methanol—4% glacial acetic acid (66/34, *v*/*v*). MeP was detected at 254 nm, while CBZ, BPA, DFC, and TCS were detected at 280 nm. Experimental data were processed using the LC Solution Lite workstation.

The TOC of the post-reaction solution was determined using a total organic carbon analyzer (Shimadzu) to evaluate the mineralization efficiency of the five PPCPs. Samples of 30 mL were collected at predetermined intervals, filtered through a 0.22 μm water-compatible filter membrane, and analyzed using the TC-IC subtraction method. Each sample was analyzed in triplicate, and the results were averaged.

Using electron paramagnetic resonance (EPR) spectroscopy, 5,5-dimethyl-1-pyrrolidine -N-oxide (DMPO) and 2,2,6,6-tetramethylpiperidine (TEMP) as spin traps, the reactive oxygen species (ROS) generated during the photocatalytic reaction were identified. The instrument parameters were set as follows: microwave frequency 9.5 GHz, microwave power 20 mW, modulation frequency 100 kHz, and sweep width 100 G.

## 4. Conclusions

In summary, this study systematically evaluated the photocatalytic degradation efficiency of five typical PPCPs (Mep, CBZ, BPA, DFC, TCS) under various light sources (UV light, simulated sunlight, and real sunlight). Under optimized reaction conditions, all five PPCPs achieved 100% complete degradation, and the total organic carbon (TOC) removal rate in the mixed pollutant system remarkably exceeded 95%. This high degree of mineralization underscores the practical potential of this technology for the complete remediation of complex medical wastewater, moving beyond simple degradation to achieve true detoxification.

Research has revealed significant differences in the apparent degradation rates of five PPCPs, with DFC exhibiting the fastest reaction rate and Mep the slowest. This fundamental disparity stems from the varying reactivity between different functional groups within each pollutant molecule and the hydroxyl radicals generated on the photocatalyst surface. The degradation rate primarily depends on the intrinsic reactivity between each pollutant molecule and hydroxyl radicals, the main active species. This reactivity is jointly influenced by the activation effect of electron-rich functional groups (-NH-, -OH) and the passivation effect of electron-withdrawing groups (-COOCH_3_, -Cl) within the molecules. Furthermore, the high efficiency of this photocatalytic system was validated under natural sunlight irradiation, demonstrating its significant potential for efficient pollutant removal without additional energy input. This study provides a robust theoretical basis and practical prospects for the efficient and sustainable treatment of complex medical wastewater containing multiple PPCPs using commercial TiO_2_ photocatalysts.

## Figures and Tables

**Table 1:** Intensity of ultraviolet light in natural light over five consecutive days.

	Radiation Intensity (mW/cm^2^)				
Date	10a.m.	12a.m.	14p.m.	16p.m.	$\bar{x}$
12.21	21.3	24.2	18.2	1.58	16.4
12.22	14.1	16.3	13.0	2.1	11.4
12.23	15.5	25.5	20.6	3.58	16.3
12.24	22.5	26.3	20.8	1.83	17.9
12.25	16.8	23.4	19.0	2.10	15.3

**Table 2:** Comparison of photocatalytic degradation and mineralization of PPCPs using different catalysts.

PPCPs	Photocatalyst	Degradation (%)	TOC Removal (%)	Ref.
Mep	MoS_2_/g-C_3_ N_4_	89.8	40.1	Duan et al., (2025) [25]
	Co/CNTs	83.2	19.2	Peng et al., (2021) [26]
	UiO-67(Zr)/g-C_3_N_4_	96.8	45.7	Bautista-Cano et al., (2024) [27]
	NH_2_-MOF-Zr@HRP	87.0	-	Zeyadi et al., (2023) [28]
	rGO/AgNPs	97.6	-	Khan et al., (2023) [29]
	CFNPs	99.9	81.7	Mmelesi et al., (2022) [30]
	Our work	100	95.0	-
CBZ	TiO_2_/BiPO_4_	38.0	-	Mohammed-Amine et al., (2025) [31]
	mAg_3_PO_4_@g-C_3_N_4_	-	87.1	Chen et al., (2024) [32]
	LaCoO_3_	100	60.0-70.0	Jing et al., (2024) [33]
	TiO_2_–C	90.0	60.0	Kubiak et al., (2024) [34]
	Our work	100	95.0	-
BPA	O-MCN	97.0	60.0	Shittu et al., (2022) [35]
	ZnO@NiFe_2_O_4_	99.9	59.2	Dehghanifard et al., (2025) [36]
	Bi_12_SiO_20_/Bi_4_O_5_I_2_/BiOBr	88.5	55.8	Xin et al., (2025) [37]
	Fe_3_O_4_@SiO_2_/PAEDTC (FSP)	94.2	85.0	Al-Musawi et al., (2025) [38]
	Fe_3_O_4_@MOF-74	95.6	-	Xiaohan Hu et al., (2024) [39]
	Our work	100	95.0	-
DFC	La-CeO_2_/ZnO	95.0	85.0	Abbadi et al., (2024) [40]
	N-TiO_2_	79.5	-	Van Tung et al., (2024) [41]
	NC/NS/rGO	65.9	-	MohammedSaleh Katubi et al., (2024) [42]
	EF	-	49.8	Romero-Espiza et al., (2024) [43]
	Mn-WO_3_/LED	-	88.0	Yazdanbakhsh et al., (2024) [44]
	Our work	100	95.0	-
TCS	O_3_/UV/ZnO	-	71.0	Topkaya et al., (2024) [45]
	TiO_2_	85.1 ± 0.490	-	Cardona et al., (2025) [46]
	Fe_3_O_4_@CuO_x_	-	86.0	Shao et al., (2022) [47]
	Our work	100	95.0	-

**Table 3:** Kinetic parameters for five PPCPs (UV lamp).

PPCPs	Kinetic Equation	Rate Constant k (min^−1^)	Correlation Coefficient R^2^
MeP	*ln*(*c*_0_/*ct*) = 0.00492x + 0.500	0.00492	0.960
CBZ	*ln*(*c*_0_/*ct*) = 0.00647x + 0.446	0.00647	0.980
BPA	*ln*(*c*_0_/*ct*) = 0.00682x + 0.426	0.00682	0.980
DFC	*ln*(*c*_0_/*ct*) = 0.02537x + 0.990	0.02537	0.970
TCS	*ln*(*c*_0_/*ct*) = 0.00652x + 0.440	0.00652	0.990

**Table 4:** Kinetic parameters of five PPCPs (xenon lamp).

PPCPs	Kinetic Equation	Rate Constant k (min^−1^)	Coefficient of Determination R^2^
MeP	*ln*(*c*_0_/*ct*) = 0.00354x − 0.118	0.00354	0.950
CBZ	*ln*(*c*_0_/*ct*) = 0.00514x − 0.144	0.00514	0.970
BPA	*ln*(*c*_0_/*ct*) = 0.00647x + 0.426	0.00647	0.950
DFC	*ln*(*c*_0_/*ct*) = 0.0110x + 0.990	0.0110	0.980
TCS	*ln*(*c*_0_/*ct*) = 0.00577x + 0.440	0.00577	0.950

**Table 5:** Kinetic parameters of 5 PPCPs (sunlight).

PPCPs	Kinetic Equation	Rate Constant k (min^−1^)	Coefficient of Determination R^2^
MeP	*ln*(*c*_0_/*ct)* = 0.0299x − 0.191	0.0299	0.950
CBZ	*ln*(*c*_0_/*ct*) = 0.0539x − 0.384	0.0539	0.950
BPA	*ln*(*c*_0_/*ct*) = 0.0342x − 0.142	0.0342	0.930
DFC	*ln*(*c*_0_/*ct*) = 0.0592x + 0.354	0.0592	0.960
TCS	*ln*(*c*_0_/*ct*) = 0.0307x + 0.00661	0.0307	0.970

## Data Availability

The original contributions presented in this study are included in the article. Further inquiries can be directed to the corresponding author.
